# Companion Animal Model in Translational Oncology; Feline Oral Squamous Cell Carcinoma and Canine Oral Melanoma

**DOI:** 10.3390/biology11010054

**Published:** 2021-12-31

**Authors:** Antonio Giuliano

**Affiliations:** Department of Veterinary Clinical Science, Jockey Club College of Veterinary Medicine, City University of Hong Kong, Tat Chee Avenue, Kowloon, Hong Kong; agiulian@cityu.edu.hk

**Keywords:** companion animal model, feline oral squamous cell carcinoma, head and neck carcinoma, immunotherapy, mucosal oral melanoma

## Abstract

**Simple Summary:**

Laboratory rodents are the most common animal models used in preclinical cancer research. Companion animals with naturally occurring cancers are an under-utilized natural model for the development of new anti-cancer drugs. Dogs and cats develop several types of cancers that resemble those arising in humans with similar clinical and histopathological features and often with similar molecular and genetic backgrounds. Exposure to environmental carcinogens, including air, food and water are also common between people and their pets. Dogs and cats are a unique model that could be integrated between the preclinical laboratory animal model and human clinical trials.

**Abstract:**

Companion animals with naturally occurring cancers can provide an advantageous model for cancer research and in particular anticancer drug development. Compared to commonly utilized mouse models, companion animals, specifically dogs and cats, share a closer phylogenetical distance, body size, and genome organization. Most importantly, pets develop spontaneous, rather than artificially induced, cancers. The incidence of cancer in people and companion animals is quite similar and cancer is the leading cause of death in dogs over 10 years of age. Many cancer types in dogs and cats have similar pathological, molecular, and clinical features to their human counterparts. Drug toxicity and response to anti-cancer treatment in dogs and cats are also similar to those in people. Companion animals share their lives with their owners, including the environmental and socioeconomic cancer-risk factors. In contrast to humans, pets have a shorter life span and cancer progression is often more rapid. Clinical trials in companion animals are cheaper and less time consuming compared to human trials. Dogs and cats with naturally occurring cancers are an ideal and unique model for human cancer research. Model selection for the specific type of cancer is of pivotal importance. Although companion animal models for translational research have been reviewed previously, this review will try to summarize the most important advantages and disadvantages of this model. Feline oral squamous cell carcinoma as a model for head and neck squamous cell carcinoma and canine oral melanoma as a model for mucosal melanoma and immunotherapy in people will be discussed as examples.

## 1. Spontaneously Occurring Cancers in Companion Animals Represent a Unique Model for Human Cancers

The main goal of cancer research is to find new diagnostic and therapeutic anti-cancer strategies. Preclinical cancer research is mainly based on laboratory animal models with most of the studies performed on tumors grown in rodents [1]. Laboratory in vitro and in vivo studies have been and remain an essential first step in cancer research. The main goal of preclinical research is to assess the toxicity and efficacy of new drugs prior to conducting human trials [2]. Despite the common use of mice and other rodents in preclinical research, these models are poorly predictive of efficacy in human clinical trials [3,4]. Despite many successful laboratory animal studies, only 5–8% of new anticancer drugs are eventually approved for clinical use [5,6].

The lack of success in preclinical laboratory mice studies, both in xenograft and genetically engineered mice, could result from the failure to accurately reproduce the biological behavior and genetic and molecular background of the artificially induced cancer compared to a spontaneous human tumor, the inability to examine the specific tumor microenvironment and host characteristics, and a lack of the complexity and heterogenicity of naturally occurring cancers [6,7]. Experiments conducted in caged laboratory animals can be affected by high levels of stress that could affect the response to investigational drugs and immunotherapy. A lack of well-defined best practice protocols for the testing, treatment and procedures performed in laboratory animals compared to human clinical trials could also introduce bias [8,9].

Companion animals represent a unique model for human cancer for various reasons. Dogs and cats, unlike laboratory rodents, develop naturally occurring cancers that closely mimic the heterogenous nature of human tumors [10,11]. Many cancers arising in dogs and cats have similar clinical signs, appearances, and biological behavior to human cancers. Microscopic appearance and genetic and molecular background are also very similar in many types of cancers in dogs, cats, and people. The outbred characteristic in dogs, compared to studies in inbred laboratory animals, provides a background of genetic diversity that more closely parallels that of humans [11,12]. In addition, compared with the murine genome, the canine genome more closely resembles the human genome [12,13].

Cancer in pets, as in people, is one of the leading causes of death [13,14]. The life span of dogs and cats has increased in recent decades and now the incidence of cancer in dogs exceeds that of people, with around 40–50% of dogs older than 10 years dying of cancer [15,16]. Companion animals share all their lives with their owners, including the environmental and socioeconomic factors that predispose to cancer development [17]. For example, obesity is considered one of the leading factors associate with increased cancer incidence, morbidity, and mortality in people [18]. Obesity also affects dogs and cats. Around 50% of dogs are considered overweight [19] and obese dogs are also more likely to have an owner that is obese [20]. Companion animals and people are exposed to similar environmental risks factors, toxins, and carcinogens such as air pollution or pesticides in food and water [21]. Spontaneous companion animal tumor models are likely to mimic the intricate and complex metabolic, genetic, and epigenetic alterations that are associated with cancer in people [22].

Companion animals have a larger body size compared to rodents, allowing for easier and more frequent blood sampling for longitudinal assessment of drug efficacy/toxicity. Furthermore, the collection of larger biopsy samples compared to those of mice can be advantageous when multiple analyses are required [23]. Identical imaging modalities can be applied to cancers in animals and humans (x-ray, CT, and MRI scan) so that any findings can be easily interpreted and compared. Responses to chemotherapy and drug toxicity in people is more comparable to those of companion animals than mice, and similar drugs are used to treat cancer in people and in pets [23]. As an example, the CHOP chemotherapy protocol involving the use of cyclophosphamide, doxorubicin, vincristine, and prednisolone is used as a standard of care for the treatment of the most common type of lymphoma, diffuse large cell lymphoma (DLCL), in both dogs and human patients. Response rates and outcomes for some cancers (approximately one year survival in dogs translates to five years in people) are also comparable [11]. However, many chemotherapy drugs are used at lower doses in pets compared to people as the main goal of treatment in veterinary oncology is to improve quality of life rather than attempting to achieve a cure [24]. While conventional chemotherapy is used at lower doses in pets to avoid severe adverse events, target therapies and immunotherapies can often be used at similar doses in pets as in human patients [25].

In the new era of cancer immunotherapy, companion animal models could play a key role in the testing and development of new treatment in people. The intricate crosstalk between the immune system and cancer is very difficult to replicate in artificially induced tumors in laboratory rodents. Dogs are exposed to a multitude of antigenic stimuli across their lifespan, including pathogenic and nonpathogenic bacteria, viruses, and parasites. Considering the large exposure of the intestine to numerous microbial antigens, it is not surprising that the intestinal microbiome can influence cancer growth and response to immunotherapy [26,27]. Dogs have a naturally developed intestinal microbiome that regulates the complex response to antigenic stimulation and diseases. Dogs are likely to respond to immunotherapy similarly to people, compared to laboratory rodents kept in disease free or minimal-disease conditions. Evaluation of the response to immunotherapy treatments and the longitudinal assessment of immune response parameters/biomarkers and side effects would be easier in companion animal than mice due to the easier collection of larger blood samples. Numerous studies in pets have assessed the relationship between cancer and the immune system; increased numbers of immunosuppressive regulatory T-cells (T-reg) have been found in various cancer types in dogs [28,29,30]. Markers of potential response to immunotherapy such as programmed cell death 1 (PD-1) and its ligand, programmed cell death ligand 1 (PD-L1), have also been found to be overexpressed in cancer cells and cancer infiltrating lymphocytes in oral melanoma, lymphoma, osteosarcoma, and urothelial carcinoma in dogs [31,32,33,34,35]. The first cancer immunotherapy vaccine has been successfully used in dogs with locally controlled oral melanoma and is now conditionally licensed in the USA [36,37]. Another new promising recombinant attenuated listeria monocytogenes vaccine expressing a chimeric human HER2 for HER-2+ osteosarcoma showed safety and efficacy in dogs and further studies are ongoing [38].

In recent years, knowledge in veterinary medicine has grown significantly. Veterinarians can specialize in various medical disciplines including companion animal oncology. Qualified veterinary oncologists, like human oncologists, perform advanced treatments and follow best practice guidelines in performing clinical trials in pets. Veterinary oncologists, similarly to their medical colleagues, conduct clinical trials in dogs and cats with a strict standardized criteria for assessing grade of toxicity and tumor response [39,40], potentially offering preclinical data that are more precise, reliable, and more likely to be translated to successful human trials than the rodent models. Dogs and cats have a short life span and cancer progresses relatively quicker than in people, allowing for a more rapid collection of end-point data such as disease-free interval (DFI) and median survival time (MST) with significantly reduced cost compared to human clinical trials [11].

Regulatory policies involving companion animal clinical trials are not always well defined and clear, but in general, the regulations are more flexible than in human clinical trials [41]. As there are no clear international guidelines, rules often vary in different countries [41]. However, it is often for the institution involved in the companion animal trial to set the rules when clear guidelines are not available [42]. Clear and detailed owner consent forms and ethical approval from the institution involved in the study are usually the main requirements to perform a companion animal clinical trial in most countries [42,43].

Despite the advantages of using companion animal models, there are few limitations that need to be considered. The cancer heterogenicity of spontaneous companion animal models is an advantage, but also a disadvantage. When specific genetic/pathway alterations need to be studied, a more homogeneous and less diverse genetic background is preferable. Another limitation is cost; despite pet trials being cheaper than their human counterparts, they are still more expensive and more time consuming than rodent preclinical studies [23]. Owner willingness to be enrolled in investigational studies and compliance with the terms and conditions of the trial are other potential disadvantages. Furthermore, an important factor that needs to be considered in pet trials is euthanasia. In many western countries, euthanasia in pets is widely recognized as a humane way to end life and so the subjective and personal decision of the owner to euthanize their pets could affect standardization of the MST (Table 1). Despite the similarities of cancers in people and companion animals, there are species-specific differences in incidence as well as biological and clinical behavior that needs to be considered before choosing a specific companion animal cancer model. Feline oral squamous cell carcinoma and canine oral melanoma have previously been considered a good model for people suffering from head and neck carcinoma and mucosal melanoma, respectively [17,44]. An updated summary of the findings in these two types of tumors for translational cancer research, including new possible translational immunotherapies, will be discussed in more detail.

## 2. Feline Oral Squamous Cell Carcinoma in Cats as a Model of Head and Neck Squamous Cell Carcinoma in People

### Incidence, Risk Factors and Biological Behavior

Feline oral squamous cell carcinoma (FOSCC) is a promising and unique model for Human head and neck squamous cell carcinoma (HNSCC) [17]. Head and neck squamous cell carcinomas (HNSCC) are the most common oral neoplasia and the sixth most common cancer worldwide, counting for 890,000 new cases and 450,000 human deaths in 2018 [45].

In HNSCC, papillomavirus is considered an important risk factor as well as tobacco smoke and alcohol consumption [45]. Cats living in households with smokers are considered at increased risk of developing FOSCC compared to non-smoking households [46,47], possibly due to the deposition of chemicals from the tobacco smoke on the coat in conjunction with feline grooming habits. Cats with a high intake of canned food in their diet and cats wearing flea collars have also been reported to be more at risk of developing SCC [46]. The relationship of papilloma virus and FOSCC is not well established. In one study, 90% of feline cutaneous SCC carried papillomavirus DNA [48]. In a recent study using next generation sequencing, the presence of feline papillomavirus in FOSCC was very low, only 1 in 20 [49]. In contrast to the situation in people, papillomaviruses are unlikely to be a risk factor for FOSCC, hence, FOSCC is likely a better model for the more aggressive HPV negative HNSCC [49].

**Figure 1:**
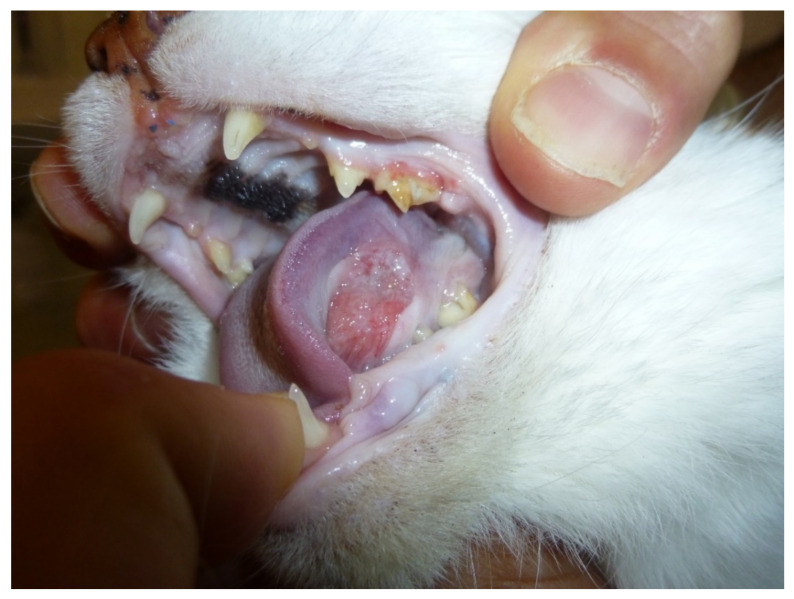
Sublingual oral squamous cell carcinoma in a 13-year-old female domestic shorthair cat.

FOSCC is a common cancer in old cats and the most common tumor affecting the oral cavity [50]. (Figure 1) FOSCC is a locally aggressive tumor with a low metastatic rate. The mucosa of the tongue, mandible and maxilla are the most common sites [51]. At presentation, metastatic rate is low, with around 14–18% having metastasized to the regional lymph nodes and around 12% to the lungs [51]. Most patients are likely to die due to the consequences of the primary tumor which impacts the ability to eat and drink [51,52]. Like FOSCC, HNSCC is a local aggressive disease with early invasion and destruction of the surrounding tissues, and metastases presenting only at a later stage [45]. Humans, like feline patients, often present at advanced stages as precancerous oral lesions are rare [45].

## 3. Molecular and Genetic Similarities

FOSCC and HNSCC have similar histological appearances with common dysregulated pathways and molecular markers [45,52,53]. P53 loss of function is frequently found in HPV-negative HNSCC and carries a worse prognosis compared with positive HPV [54]. Mutation of p53 is commonly found in FOSCC [47] and seems to be associated with tobacco smoke exposure [47].

The epithelial growth factor (EGF) signaling pathway involved in the cancer development, progression, metastasis, and angiogenesis, is dysregulated in many cancers [55]. EGF receptor (EGFR) overexpression has been found in both FOSCC and HNSCC [53,56]. Increased expression in people with HNSCC has been associated with poor survival, and anti-EGFR monoclonal antibodies have been used with limited success [55]. EGFR is overexpressed in a large proportion of FOSCC, but the prognostic significance is still controversial [17].

COX-2 expression is mainly involved in tumor development, growth, and neo angiogenesis. High Cox-2 expression is found in a large proportion of HNSCC, and overexpression is considered a negative prognostic factor [57]. In FOSCC, expression of COX-2 is variable, with different studies reporting between 18 and 60% of expression [58,59,60]. It is not clear if COX-2 expression in FOSCC is of prognostic significance, but improved survival has been found in cats treated with COX-2 inhibitors [61].

Overexpression of VEGF has been found in FOSCC and HNSCC. Increased expression can be correlated with poor prognosis in people [62,63] and in one study in cats, and COX-2 and VEGF expression were correlated with FOSCC disease progression [64].

STAT3 phosphorylation and hyperactivation has been found in HNSCC and FOSCC and it has been associated with a poor prognosis in people [65,66]. In FOSCC, cell lines phosphorylated STAT show high levels of expression and treatment, with a STAT inhibitor producing a significant biological effect [66]. STAT inhibition could be a new target treatment for both FOSCC and HNSCC.

WNT signaling is a signal transduction pathway known to be dysregulated in many human cancers [67]. Oncogenic signaling by the WNT–β-catenin pathway contributes to HNSCC, and overexpression and dysregulation of the WNT- β-catenin pathway has been found in HNSCC [68,69,70]. Similarly, putative targets of WNT signaling transduction were found to be upregulated in FOSCC [52].

BMI1 (B cell specific Moloney murine leukemia virus integration site 1) is an important biomarker of cancer stem cells (CSC). CSC are involved in cancer transformation, progression, and metastasis. High levels of BMI-1 have been found in both FOSCC and HNSCC with possible prognostic implication and future new treatment avenues in both cancers [71,72].

## 4. Therapeutic Strategies

Despite the progress made over the past two decades, HNSCC remains a tumor with high mortality rates due to frequent late-stage presentations, and more effective systemic treatments are needed [45].

The main treatment modalities for both FOSCC and HNSCC are surgery and radiotherapy with systemic treatment achieving only modest results [51,73]. Treatment for advanced FOSCC with standard chemotherapies have been unsuccessful, as tumor response and/or improved survival time are rarely achieved [51]. Similarly, in people, standard chemotherapy treatment with carboplatin, with or without fluorouracil, is quite disappointing for advanced tumors [73]. Tyrosine kinase inhibitor treatments have been used in both advanced FOSCC and HNSCC. In advanced HNCC, for example, the use of EGFR inhibitor Gefitinib has shown only a very modest clinical efficacy [74]. Similarly to feline patients, the use of a multi-kinase inhibitor Toceranib has produced only minor survival improvement in FOSCC cases [75].

The prognosis for cats with FOSCC is often very poor, with cats treated palliatively with COX-2 inhibitors and/or other pain management surviving only 2–3 months [61] while cats treated with surgery and radiotherapy only 3–5 months [76,77]. Similarly, the prognosis for advanced stage HNSCC (stage -III-IV) is very poor, with most people dying in less than one year [73].

In conclusion, at a microscopic and macroscopic level and from molecular background to clinical behavior, FOSCC and HNSCC share interesting similarity [78]. FOSCC could be a good model for new anti-cancer drug trials for advanced HNSCC. The high incidence of FOSCC, and the lack of effective treatment, translate into fast clinical trials in cats compared to people. Endpoints like DFI and MST are reached quickly as most of the cats with only palliative treatment die or are euthanized in 2–3 months [61]. As no standard of care or effective drugs are available, many FOSCC patients could be potential candidates, and a high number of enrolled patients would translate into quickly achievable results. However, when considering owned feline patients, there are some potential problems that need to be taken into consideration, such as owner willingness to enroll the cat in an investigational study, owner compliance with the terms and conditions of the trial, and the potential extra costs for the owners. For studies involving frequent administration of oral drugs, willingness and/or capability of the owner to administer tablets/capsules, palatability of the compound or the possibility of oral pain when opening the mouth will need to be taken into consideration.

## 5. Canine Oral Melanoma as a Translational Model of Mucosal Melanoma and Immunotherapy in People

### Biological Behavior and Molecular Similarities

Canine oral melanoma might represent a unique model to study mucosal melanoma in people, and to assess the safety and efficacy of new immunotherapy drugs before human clinical trials.

**Figure 2:**
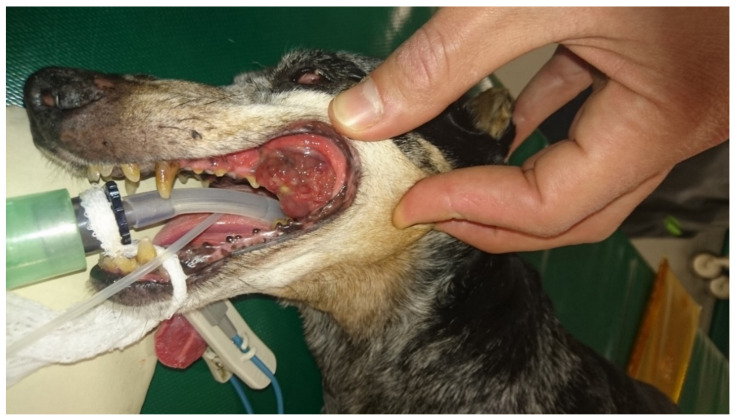
Mucosal melanoma of the oral cavity in a 9-year-old mixed breed dog.

Canine melanoma is a very common tumor in old dogs and the most common malignant tumor of the oral cavity [79] (Figure 2). The tumor originates from the neoplastic transformation of resident melanocytes of the oral cavity. Human mucosal melanoma is a rare cancer that affects mainly the oropharyngeal and nasal cavity [80]. The etiopathogenesis of human mucosal melanoma, in contrast to that of cutaneous melanoma, is largely unknown. The lack of exposure to UV-light rules this out as a causal factor for mucosal melanoma [81]. The risk factors and etiopathogenesis of oral melanoma in dogs are also unknown, but a genetic predisposition has been hypothesized due to the predisposition of some small breeds with heavily pigmented oral mucous membranes [82]. Mucosal melanomas in dogs are locally aggressive tumors with a high rate of metastasis especially to the loco-regional lymph nodes and lungs [79,83]. Contrary to people, canine cutaneous melanoma or melanocytoma are usually benign lesions often cured by a complete surgical excision [83]. In people, mucosal melanoma is much less common compared with dogs but shares similar aggressive biological behavior [44].The prognosis for mucosal melanoma in people is poor, with only around 20–30% of patients alive at five years [80,84]. Similarly, in dogs, advanced stage oral melanoma carries a very poor prognosis with survival ranging from two to five months [79,82,83]. Mucosal melanoma in dogs and people share a similar histopathological appearance and molecular/genetic background [44]. Oncogene mutations commonly found in cutaneous melanoma in humans, like BRAF and NRAS, are uncommon in mucosal melanoma in either humans or dogs, while activation of the ERK and AKT signaling pathways are common in both species [44]. Aberrant expression of the oncogene KIT and mutation of platelet derived growth factor receptor, PDGFRA, are more common in mucosal melanoma compared to cutaneous melanoma in people [85]. In canine oral melanoma, KIT mutation is uncommon and anti-KIT targeted therapy has resulted in only modest results [49,86]. However, PDGFRα/β expression was found in around 50% of oral canine melanoma and α and β co-expression was shown to correlate with a worse prognosis [87].

## 6. Immunotherapeutic Strategies

The most common treatment for mucosal melanoma in both dogs and human patients is surgery and radiotherapy with systemic chemotherapy treatment rarely effective in both species [88,89,90,91]. Effective systemic treatments for the long-term control of advanced cancer stages in both people and dogs are lacking. BRAF tyrosine kinase inhibitors (TKIs) have been successfully used in people with cutaneous melanoma, but due to the low frequency of BRAF mutation in mucosal melanoma, TKIs are rarely effective in this type of melanoma [92].

It is well-known that cancers use various mechanisms to inhibit the anti-cancer immune response and induce immunotolerance. Immune checkpoints and programmed cell death in T-lymphocytes and the respective ligands PD-L1 in cancer cells, all play a pivotal role in cancer-associated immune suppression and immune evasion [93]. High expressions of PD-1/PD-L1 have been proposed as cancer markers to predict response to treatment to monoclonal anti PD-1/PD-L1 checkpoint inhibitors, and expression of PD-1 and PD-L1 have been found in cancer cells and tumor infiltrating lymphocytes in both canine and human melanoma [30,31]. Other recognized markers of cancer immune evasion like a high number of T-regulatory cells, increased expression of cytotoxic T lymphocyte antigen-4 CTLA-4, and increased lymphocytes activation inhibitor indoleamine-pyrrole 2,3-dioxygenase IDO, have been found in canine melanoma and were found to correlate with a worse prognosis [94].

Tumor-associated antigens (TAAs) are antigens expressed in tumor cells that are not present in normal tissue cells, or expressed at a higher level in cancer cells [95]. TAA are responsible for immune responses to cancer and numerous TAAs have been discovered in various cancers including melanoma [96]. Melanoma-associated antigens are often differentiation antigens, antigens derived from specific proteins expressed in melanoma and normal melanocytes, and are involved in melanin biosynthesis or melanosome biogenesis [97,98]). The most studied melanoma antigens (MAA) are tyrosinase, gp100/pmel17, and Melan-A/MART-1 [97,99,100]. MAA have been used to develop an anticancer vaccine in dogs that could be translated to vaccine immunotherapy in people [101,102,103].

Cancer immunotherapy has recently gained momentum due to the success of immunotherapy treatments for a subset of cancers and, in particular, cutaneous melanoma. The success of the anti PD-1 monoclonal antibody in mucosal melanoma in people has been encouraging, but survival compared to cutaneous melanoma remains very poor [104]. Recently in dogs with oral melanoma, immunotherapy with an anti PD-1 monoclonal antibody has also achieved some promising results [105,106]. Immunotherapy with a xenogeneic human tyrosinase DNA vaccine is already available for dogs with stage II and III locally controlled oral melanoma and it shows a significant efficacy in a subset of patients [36,107].

Another melanoma antigen called chondroitin sulphate proteoglycan-4 (CSPG-4) has been used to develop a canine vaccine. CSPG-4 is a transmembrane protein that plays an important role in cell proliferation, adhesion, migration, and survival [108,109]. It participates in signaling transduction, presenting and linking growth factors to the extracellular matrix (ECM), and enhancing growth factor activity and integrin-mediated pathways [110,111]. CSPG4 is overexpressed in various cancers including melanoma in dogs and human patients [112,113]. Recently, vaccination immunotherapy with a human chondroitin sulphate proteoglycan-4 (hCSPG4) DNA-based vaccine delivered by intramuscular injection followed by electroporation has shown some efficacy in canine oral melanoma after surgical resection [114,115]. In human patients, various anti-cancer vaccines have entered clinical trials, but none have been approved due to a lack of significant cancer response or increase in survival time [116]. However, human trials are often performed in people with an advanced cancer stage [117,118], while anti-cancer vaccines are likely to be more beneficial in patients with microscopic or at early stage of disease [119]. This is mainly due to the time required for the patient to mount an effective immune response to the cancer vaccine, causing a delayed anti-cancer effect with a clinically detectable response that can require weeks or months to be achieved [119]. A successful vaccination strategy in dogs could positively translate in human trials with early disease, and a combination of vaccine with check point inhibitors could also achieve better results [120]. Due to the difficulty in recruiting large numbers of human patients with mucosal melanoma, the large number of available dogs with a similar disease make canine oral melanoma an interesting model with which to investigate new treatment strategies.

Another immunotherapy strategy that could take advantage of the canine oral melanoma model is adoptive cell transfer (ACT) treatment. With this approach, antigen-specific T-cells are isolated from the patient, expanded, activated in vitro, and reinfused into the same patient. One promising ACT treatment is the use of CAR-T cell, where T lymphocytes are genetically modified to express chimeric antigen receptors that recognize a specific surface tumor antigen. CAR -T cells have the main advantage of being non-MHC II restricted, so they can be administered from healthy allogenic donors, and they are still effective in cancers with down-regulation of MHCII [121,122,123]. However, despite the success of CAR-T CD19 cell therapy in lymphoproliferative diseases, the results in solid tumors, including melanoma, have been disappointing [124,125]. In melanoma, CAR-T cell therapy has shown success in preclinical models, but success in clinical trials has not been satisfactory [125,126]. It is now clear that mouse models are probably not a good model to predict response and side effects to immunotherapy in people [126]. Major drawbacks of CAR-T cells in solid tumors are the difficulty of finding specific antigens that are widely expressed in the cancer and not expressed in normal tissues, a lack of homogeneous penetration of the CAR-T cell in solid cancer tissue (due to increased tumoral interstitial pressure, hypoxic and immune suppressive environment, and thick extracellular matrix), off-target effects with significant adverse events, and development of resistance [124,127].

ACT therapy has not yet been used in canine oral melanoma, but it is feasible in canine patients, and it has already been investigated in dogs with lymphoma and osteosarcoma. This approach not only appears to be promising, but also appears to be better compared to murine models with regards to studying the response and side effects to immunotherapy [128,129,130]. As many MAA are shared between human and canine melanoma, evaluation of new antigen specific adoptive cell therapy could be used in dogs as a more efficient preclinical model than mice, before starting a clinical trial in people.

In recent years, the role of intestinal microbiota has been found to a play a key role in health and disease and the fecal microbiome has been found to affect cancer growth and even response to immunotherapy [26,27]. In a recent small human clinical trial, a fecal microbiota transplant showed some efficacy in restoring response to patient refractory to anti-PD-1 monoclonal antibodies [131]. Interestingly, dysbiosis and alteration of the microbiome have been found in dogs with cancers [132,133] and fecal transplantation has been investigated in dogs with inflammatory bowel disease (IBD and refractory IBD with some success [134,135]). Immunotherapy in combination with fecal transplantation could be beneficial in both species and canine patients could be a useful model to test for this or other types of combined immunotherapy approaches that could be translated to people. Despite the significant advantage of this canine model, there are similar limitations as discussed in relation to FOSCC, including owner compliance with the terms and conditions of the trial, potential extra costs for the owners, and willingness and/or capability of the owner to administer tablets/capsules, all which need to be taken into consideration.

## 7. Conclusions

FOSCC and canine oral melanoma are only two examples of how cancers in pets could be used as a translational model for the development of new anti-cancer treatments in people. The collaboration and sharing of knowledge between scientists working in preclinical research, veterinary oncologists, and human oncologists should be implemented in a “one health” “one oncology” approach. Integration of companion animal clinical trials between laboratory preclinical studies and human clinical trials could improve the bench-to-bedside success rate of new anticancer drugs development.

## Figures and Tables

**Table 1:** Advantages and disadvantages of companion animal models for translational cancer research.

Advantages	Disadvantages
⮚ Naturally occurring cancers ⮚ Cancer heterogenicity ⮚ Shared environmental and socioeconomic factors ⮚ Similar tumor histopathology, molecular and genetic background ⮚ Large body size ⮚ Similar biological behavior ⮚ Similar response to treatment and side effects ⮚ Similar cancer staging and imaging modalities ⮚ Intact and functional immune system ⮚ Pets’ clinical trials are cheaper than human trials ⮚ Standardization in reporting drug toxicity and cancer response ⮚ End-point measurements (DFI/MST) * in pets are reached faster than in human trials	⮚ Tumor heterogenicity ⮚ Longer studies and higher costs compared to mouse model ⮚ Different incidence for some cancer types in human and pets ⮚ Owner request for euthanasia can affect MST * measurement ⮚ Owner compliance

* DFI disease free interval, MST median survival time.
